# Evaluating the feasibility of a group psychosocial intervention for migrant and host community women in Ecuador and Panamá: protocol for a multi-site feasibility cluster trial

**DOI:** 10.1186/s40814-022-01085-1

**Published:** 2022-06-15

**Authors:** M. Claire Greene, Annie Bonz, Maria Cristobal, Carolina Vega, Lena S. Andersen, Alejandra Angulo, Andrea Armijos, María Esther Guevara, Lucia Benavides, Alejandra de la Cruz, Maria Jose Lopez, Arianna Moyano, Andrea Murcia, Maria Jose Noboa, Abhimeleck Rodriguez, Jenifer Solis, Daniela Vergara, Jodi Scharf, Priya Dutt, Milton Wainberg, Wietse A. Tol

**Affiliations:** 1 Program on Forced Migration and Health, Heilbrunn Department of Population and Family Health, Columbia University Mailman School of Public Health, New York City, USA; 2 HIAS, Silver Spring, USA; 3 HIAS, Quito, Ecuador; 4 Global Health Section, Department of Public Health, University of Copenhagen, Copenhagen, Denmark; 5 HIAS, Panamá City, Panamá; 6 Department of Psychiatry, Columbia University/New York State Psychiatric Institute, New York City, USA

**Keywords:** Psychosocial wellbeing, Psychosocial intervention, Community-based, Humanitarian emergencies

## Abstract

**Background:**

Community- and strengths-based psychosocial interventions are central to mental health and psychosocial support guidelines, but rigorous evidence regarding the effectiveness of these interventions is limited. The complexity and variability that is inherent to many community-based psychosocial interventions requires innovative strategies in order to facilitate the comparability and synthesis across research studies without compromising the fit and appropriateness of interventions to specific study populations and context. *Entre Nosotras* is a community-based psychosocial intervention developed for migrant and host community women that is designed to be flexible enough to enable integration of external intervention components and adaptable to diverse study contexts and populations. This protocol describes a study that aims to evaluate the appropriateness, acceptability, and feasibility of integrating a standardized stress management intervention into *Entre Nosotras*.

**Methods:**

This study will evaluate the appropriateness, acceptability, feasibility, and safety of intervention and research procedures for a cluster randomized comparative effectiveness trial conducted in Ecuador and Panamá with migrant and host community women. In this feasibility trial, we will allocate communities nested within the three study sites to the integrated *Entre Nosotras* + stress management intervention versus *Entre Nosotras* alone through stratified randomization. Migrant and host community women residing in these study communities who report low to moderate levels of distress will be allocated to the intervention condition that their community is assigned (*n* = 220 total). We will collect quantitative measures of psychosocial wellbeing, psychological distress, coping, social support, and functioning from study participants. We will collect quantitative measures of fidelity and facilitator competencies through observation and facilitator self-assessment. Data on appropriateness, acceptability, feasibility, and safety will be gathered from participants and facilitators through quantitative assessments at 0, 5, and 10 weeks post-enrollment and qualitative interviews conducted with all facilitators and a subset of 70 study participants during the post-intervention follow-up period.

**Discussion:**

Results from this feasibility trial will determine whether a multi-site cluster randomized comparative effectiveness trial of an adaptable community-based psychosocial intervention for migrant and host community women is relevant, acceptable, and feasible.

**Trial registration:**

ClinicalTrials.gov identifier: NCT05130944. Registered November 23, 2021—retrospectively registered.

## Background

By the end of 2020, over 80 million people were forcibly displaced due to persecution, conflict, violence, human rights violations, and other emergencies globally [1]. Displaced populations face increased exposure to potentially traumatic events and ongoing adversity that increase their risk of mental health and psychosocial problems [2–4]. The evidence supporting the effectiveness of mental health and psychosocial support (MHPSS) programs in humanitarian settings has grown over the past few decades [5, 6]. However, existing evidence is primarily available for focused psychological interventions to treat mental health problems adapted from interventions developed in high-income contexts as opposed to promotion and prevention programs designed within the target population that focus on mitigating risk of mental health problems and enhancing psychosocial wellbeing. Furthermore, few MHPSS interventions have focused on the social dimension of mental health and psychosocial wellbeing [5]. Social difficulties such as isolation, discrimination and xenophobia, and lack of community support and connectedness are often salient problems affecting migrant communities [7, 8]. These outcomes are rarely included as MHPSS intervention targets or outcomes in humanitarian settings.

Community- and strengths-based psychosocial interventions are central to MHPSS recommendations in humanitarian settings despite remaining underrepresented in existing research evidence [6, 9, 10], and are considered a critical research priority [11]. Available evidence from evaluations of psychosocial prevention and promotion interventions is heterogeneous with respect to the types of interventions and evaluation approaches, creating challenges for comparability and evidence synthesis [6, 12]. Researchers have argued that in order to advance the evidence on psychosocial interventions there needs to be better specification of interventions to enable replication, design and tailoring of psychosocial interventions for specific subgroups (e.g., women) and subgroup-specific outcomes, and rigorous evaluations of community-based interventions that enhance psychosocial wellbeing through improved social and community-related processes [5, 6, 13].

In the proposed multi-site study, we aim to examine the feasibility of a community-based psychosocial intervention for women using an adaptable, but manualized process to balance the need to ensure fit with the need for replicability in existing research on psychosocial interventions [14]. The intervention was designed to be adaptable by specifying the components of the intervention that are fixed and those that are adaptable. The adaptable components are modifiable activities that can be substituted with more culturally or contextually relevant activities that are expected to achieve the same objective as the original activity. This study proposes to use a comparative effectiveness design that enables the intervention components targeting social processes, which we hypothesize may require more tailoring to fit the socio-cultural context, to be adaptable across settings while testing the integration of a standardized stress management component on psychosocial wellbeing for women in three diverse contexts. A comparative effectiveness design utilizing an equal attention control also minimizes the risk of information bias that threatens the validity of many wait-list controlled trials of psychosocial interventions (e.g., due to placebo effects).

The proposed research aims to address major gaps in evidence on MHPSS interventions and incorporates processes to promote local adaptability and fit without compromising the intervention rigor. A community-based approach that combines testable, yet locally adaptable components to improve mental health, coping, functioning, social support and connectedness, and a sense of safety is needed to effectively promote the wellbeing of displaced women affected by chronic adversity and humanitarian emergencies.

### Objectives

The purpose of the current protocol is to describe a feasibility cluster randomized trial examining the integration of a scalable stress management intervention into *Entre Nosotras* (‘among/between us’), a community-based psychosocial intervention for migrant and host community women in Ecuador and Panamá. The two study conditions include (1) *Entre Nosotras*, a flexible and adaptable women’s group intervention designed to mobilize social support, strengthen community connectedness, and stimulate collective action to promote the safety and wellbeing of women (standard intervention); to (2) *Entre Nosotras* with an additional scalable stress management intervention (enhanced intervention). The objective of this feasibility trial is to examine the appropriateness, acceptability, feasibility, and safety of *Entre Nosotras* as well as the integrated stress management component and to collect data to inform the design of a definitive cluster randomized comparative effectiveness trial.

## Methods/design

### Study setting

Latin America has recently experienced increasing levels of forced migration due to political conflict, economic crises, climate-related events, community violence, and other factors. Panamá hosts refugees and migrants from the Northern Triangle (Guatemala, Honduras, El Salvador), Nicaragua, Colombia, and Venezuela, among other countries [15]. Ecuador has long been a host country for displaced persons from Colombia and, more recently, Venezuela [16]. Migrants in these contexts face mental health and psychosocial problems, protection risks, and disrupted social and community support systems [17]. Gaps in the provision of psychosocial services, despite the high prevalence of psychosocial problems and threats to the safety and wellbeing of refugees and migrants, persist in both countries and have been exacerbated due to the COVID-19 pandemic [18].

The proposed research will be conducted in three sites where the implementing partner, HIAS, an international non-governmental refugee protection organization [19], is currently providing services to migrants in Ecuador (Guayaquil and Tulcán) and Panamá (Panamá City area). Guayaquil is the largest city in Ecuador located in the coastal region and is a destination for many migrants from Colombia and Venezuela. Tulcán is located in the highlands of Ecuador on the border with Colombia and is primarily rural and often a temporary place of transit for migrants. Panamá City is the capital of Panamá and a destination for migrants, primarily from Central and South America. Many migrant communities live in peri-urban areas surrounding Panamá City. The majority of migrants in Panamá and approximately half of migrants in Ecuador are female, most of whom are of reproductive age [20]. These three sites were selected because they have large and increasing populations of forced migrants and are diverse in terms of urbanicity, service delivery systems, populations, and other implementation factors.

### Study design

We will conduct a feasibility cluster randomized trial and mixed-methods process evaluation in nine communities across the three study sites. The randomization procedure will be stratified by site. Within each of the three sites, we will randomly allocate at least half of the communities to receive the integrated intervention (*Entre Nosotras* with a standardized stress management component) as compared to *Entre Nosotras* alone. A definitive trial with this design will allow us to examine whether adding a structured stress management component to *Entre Nosotras* enhances study outcomes beyond any effects derived from the *Entre Nosotras* intervention alone. This design also allows for flexibility in the specific activities within the community-based women’s group (*Entre Nosotras*) component to be tailored to the local population and context in each of the three study sites and may serve as a model for future research balancing replicability and fit.

### Sequence generation

Communities (clusters) will be allocated to study condition using a random number generator in Stata by a researcher not affiliated with the project.

### Allocation concealment mechanism

Since this is a cluster-randomized design there will be no allocation concealment mechanism.

### Blinding

Only feasibility trial participants will be blinded to the conditions of the two arms.

### Intervention

*Entre Nosotras* is a community- and strengths-based intervention designed to mobilize social support, strengthen community connectedness, and stimulate collective action to promote the safety and wellbeing of women. The intervention was selected and designed through a formative qualitative research and community consultation process, which is described in detail within a forthcoming publication. Through this formative research process, we identified social problems (e.g., interpersonal violence, xenophobia and discrimination, social isolation, and loneliness) and psychological problems (e.g., emotional distress, sadness) to be among the most salient problems affecting migrant women in these communities. Through a community consultation and design process, women recommended a dynamic and interactive group intervention that mobilized social support and leveraged community resources, while also providing skills to help cope with psychological distress and improve safety. With this information, we designed *Entre Nosotras* to target the social problems and processes that we discovered during this formative research and further identified a standardized, stress management intervention for coping with psychological distress that could be integrated to enhance the *Entre Nosotras* components.

The intervention includes five weekly sessions lasting approximately 2 to 3 h each delivered by female facilitator pairs within the community. The intervention is based on the HIAS Mental Health and Psychosocial Support Curriculum [21], principles of Psychological First Aid [22–24], and the Community Action Cycle [25], which is intended to generate community-led problem solving around priority issues affecting participants’ wellbeing. The sessions include a series of fixed and modifiable components. The fixed components of the intervention are the scaffolding of the intervention that introduce the topics for each session and guide the facilitator through the session (see Table 1). These fixed components include specific objectives, brief facilitator scripts, and tips for the facilitator, including possible adaptations for remote delivery. The modifiable components make up the majority of the intervention and include suggested activities that align with the session objectives. The program implementers/managers are encouraged to substitute these suggested activities for ones that are culturally relevant and expected to achieve the specified objectives. This adaptable manual is designed to maintain consistency around the objectives and concepts that are covered within the sessions (i.e., function), while allowing for variability in how these concepts are expressed and engaged with in groups (i.e., form).

**Table 1:** Overview of the intervention

Session	Objectives
1. Building trust, security, and connection	• Build trust and group identity • Collectively set group expectations, rules, and goals • Introduce concepts of coping and grounding (in enhanced condition only)
2. Psychosocial wellbeing	• Define the concept of psychosocial wellbeing and its dimensions • Describe the factors that contribute to psychosocial wellbeing, including the impacts of adversity • Practice grounding as a coping skill (in enhanced condition only)
3. Gender, gender-based violence, and safety	• Reflect on concepts related to gender, wellbeing, and protection • Identify resources to promote the safety and wellbeing of women • Introduce unhooking from difficult thoughts and feelings as well as acting according to your values as coping skills (in enhanced condition only)
4. Strengths and psychosocial resources	• Identify social and psychological strategies and resources to support safety and psychosocial wellbeing • Mobilize peer support • Revisit acting on your values and introduce being kind as coping skills (in enhanced condition only)
5. Community resources	• Identify community resources that promote protection, safety, and wellbeing • Strengthen community support networks • Introduce making room as a coping skill (in enhanced condition only) • Generate a group support and action plan for the future

For example, in Latin America there is a long history of using theater to generate collective discussion and action around social issues [26]. In session 4, *Entre Nosotras* uses this ‘theater of the oppressed’ methodology to generate strategies to support women who are affected by family problems/violence, community tension, and xenophobia. However, in other contexts, there may be more culturally appropriate approaches to generate discussion and collective action on sensitive social issues, which can be used to substitute the theater of the oppressed activity. Additionally, within this study there were notable differences in the intervention across the study sites. For example, in Ecuador, the intervention concludes with a *pamba mesa*, which is a traditional communal meal that symbolizes social solidarity. In Panamá, the final session closes with the participants sharing a small gift (e.g., crafts, bracelets) made for another member of the group along with a discussion of what they gained from the intervention and how they plan to sustain the group. They are also invited to bring something to share with the group that, similarly to *pamba mesa*, may include food. Each of these activities shares the same function (e.g., reinforcing social connectedness and sustained group identity), but applies a form that fits the local context and traditions.

The *stress management* component that we have integrated into the *Entre Nosotras* intervention for the experimental condition is based on the World Health Organization’s Doing What Matters in Times of Stress intervention [27], a self-guided stress management intervention that is intended to provide practical skills to help people cope with psychological distress. It includes an illustrated guide along with optional audio exercises that are based on Acceptance and Commitment Therapy (ACT), a type of cognitive behavioral therapy that incorporates mindfulness approaches to develop skills for managing difficult thoughts and feelings [27, 28]. Doing What Matters in Times of Stress includes five skills-based tools: (1) grounding; (2) unhooking from difficult thoughts; (3) acting on your values; (4) being kind; and (5) making room [27]. As shown in Table 1, we distributed activities to support the development of these skills across the five sessions. Within the sessions, the facilitators provide an overview of the skill(s) and the participants practice the skill at least once during the session with the support of audio exercises developed and made publicly available by the World Health Organization in thirteen languages (https://cdn.who.int/media/docs/default-source/mental-health/doing-what-matters-audio-audio-links-in-different-languages.pdf?sfvrsn=1f449f8d_16). Each session ends with homework that involves setting a goal for practicing the skill, which is revisited during the check-in at the beginning of the following session.

### Participants and procedures

#### Study eligibility

The target population for this study includes Spanish-speaking adults who identify as women, reside in the study community, and are willing and able to engage in the intervention. We aimed to recruit a mix of both migrant and host community women. We plan to recruit 70–80 women from each of the 3 sites (*n* = 220 women total; *n* = 20–40 women per community) into the feasibility trial. The eligibility criteria are as follows:

#### Inclusion criteria

We will include adult (18+ years) women residing in the study community who speak and understand Spanish.

#### Exclusion criteria

We will exclude participants if they report severe psychological distress (Kessler-6 score > 13), disclose suicidal thoughts or feelings, or display signs of cognitive impairment that would prevent participation in a group psychosocial intervention during the screening interview. Participants who are excluded for these reasons will be referred to appropriate services within HIAS or through partner implementing organizations.

A subset of participants from the feasibility trial (*n* = 10 per community, or until we reach saturation) as well as all intervention facilitators will be selected to complete qualitative semi-structured interviews after the intervention period. Participants will be purposively selected using maximum variation sampling to represent a range in level of engagement, level of distress, among other factors that emerge as important contributors to implementation success.

### Recruitment, screening, and assessment procedures

All participants will be recruited through referral from HIAS staff, community outreach workers, and community leaders. Interested individuals will be connected by phone or in person with members of the research team, who will provide further information about the study. After providing further information, research assistants will invite interested individuals to complete a brief screening assessment. Verbal consent must be provided prior to commencing the screening. We will obtain written informed consent from eligible participants prior to administering a baseline assessment. Within 2 weeks of the baseline assessment, participants will attend the first session of the *Entre Nosotras* group to which their community was randomly allocated (enhanced vs. standard condition). Follow-up assessments will be conducted by a research assistant within 1-week post-intervention and 5 weeks post-intervention. Facilitators and participants selected to participate in the process evaluation will be invited to participate in a qualitative interview occurring within the post-intervention follow-up period (i.e., between end of intervention and 5 weeks post-intervention).

Assessments are designed to be conducted remotely or in person, depending on COVID-19 policies and recommendations. Participants who complete the assessments in person will be provided with reimbursement for their transportation costs and a take-away snack as an incentive for their participation. Participants who complete the assessments remotely will be provided with airtime or connectivity reimbursement for their participation.

### Outcomes

This feasibility trial aims to assess the appropriateness, acceptability, feasibility, and safety of implementing and evaluating *Entre Nosotras*, including when integrated with a stress management component, for migrant and host community women in Ecuador and Panamá. These outcomes will determine whether the study should progress to a definitive cluster-randomized comparative effectiveness trial and whether any research design or intervention adaptations are needed. These feasibility trial outcomes will be assessed using indicators and means of verification that are measured at the participant, service, and implementation level [29]. Table 2 details the outcomes, indicators that will determine progression to the definitive trial, means of verification, and assessment time points.

**Table 2:** Study outcomes, indicators, and means of verification

Outcome	Indicator(s) for progression to definitive trial	Means of verification	Participants assessed	Time points			
				Screening/baseline	Intervention ***(5 weeks)***	Post-intervention	5-week follow-up
Appropriateness	**Eligibility:** > 50% of persons are screened are eligible	Routine study monitoring forms	Screened individuals	X			
	**Sensitivity to change:** Small to moderate within-group changes in outcome measures (*d* > 0.2)	Brief-COPE, K-6, OSS, PWI, WHODAS	Participants	X		X	X
	**Intervention fidelity:** > 75% fidelity to the intervention elements	Fidelity assessment^a^	Facilitator pairs		X		
	**Stakeholder perspectives:** Participants and facilitators report that the intervention and research procedures are appropriate and relevant to the needs of host and migrant women	Qualitative interviews	Participants, facilitators			X	X
Acceptability	**Intervention engagement and completion:** > 85% of participants attend first session; > 67% participants complete at least 4 sessions	Routine study monitoring forms	Participants		X		
	**Intervention usability:** Facilitators consider the intervention to have above average usability (IUS Score > 68).	Intervention Usability Scale	Facilitators		X		
	**Stakeholder perspectives:** Participants and facilitators report that the intervention and research procedures are acceptable and safe for host and migrant women	Qualitative interviews	Participants, facilitators			X	X
Feasibility	**Recruitment rate:** Average rate of enrollment is 5 women per week, at minimum, in each community	Routine study monitoring forms	Participants	X			
	**Randomization:** No moderate to large differences (*d* > 0.5; Φ > 0.3) in baseline characteristics between study conditions within study sites	Demographics, Brief-COPE, K-6, OSS, PWI, WHODAS	Participants	X			
	**Attrition:** > 80% of participants complete baseline, post-intervention, and follow-up assessments	Demographics, Brief-COPE, K-6, OSS, PWI, WHODAS	Participants	X		X	X
	**Fidelity to research procedures:** No major protocol deviations	Routine study monitoring forms	Research assistants	X	X	X	X
	**Facilitator competencies:** An average score of > 2 on competency items, suggesting partially-fully demonstrating the competencies during intervention implementation	ENACT^a^	Facilitators		X		
	**Contamination:** Stress management components from the experimental conditions are included in all sessions for groups randomized to the enhanced intervention and none of the sessions for groups randomized to the standard intervention	Fidelity assessment^a^	Facilitator pairs		X		
	**Performance of outcome measures:** All outcome measures display adequate construct validity and internal consistency	Brief-COPE, K-6, OSS, PWI, WHODAS	Participants	X		X	X
	**Stakeholder perspectives:** Participants and facilitators report that the intervention and research procedures are feasible, adoptable, scalable, and sustainable. Participants and facilitators identify barriers and facilitators to implementation as well as strategies for overcoming/leveraging these factors.	Qualitative interviews	Participants, facilitators			X	X
Safety	**Adverse events:** Detected in < 10% of participants; no serious adverse events attributed to study participation	Adverse event reporting	Participants	X	X	X	X
	**Stakeholder perspectives:** Participants and facilitators report that the intervention and research procedures are acceptable and safe for host and migrant women	Qualitative interviews	Participants, facilitators			X	X

*Abbreviations*: *IUS* Intervention Usability Scale, *K-6* Kessler-6, *OSS* Oslo Social Support Scale, *PWI* Personal Wellbeing Index, *WHODAS* World Health Organization Disability Assessment Schedule

^a^Fidelity assessment includes a self-administered assessment completed by the facilitator pair at the end of each session as well as an external assessment completed by a member of the research team at two or more sessions per group. Six items from the ENACT that were relevant for a non-clinical, group psychosocial intervention (non-verbal communication and active listening; verbal communication; rapport building and self-disclosure; exploration, interpretation and normalization of feelings; demonstration of empathy, warmth, and genuineness; elicitation of feedback when providing advice, suggestions, and recommendations) were selected to assess facilitator competencies during the facilitator training and are also evaluated by a member of the research team at the 2+ sessions during which they administer the external fidelity assessment

### Participant-level measures

Eligible participants will complete assessments administered by a member of the research team at baseline, within 1-week post-intervention, and 5 weeks post-intervention. These assessments include the following measures that will be evaluated for their suitability as participant effectiveness outcomes in a definitive trial. We used existing Spanish translations when available. For measures that required translation, we had a bilingual member of our team with experience working with the target population prepare the initial translation, which were then reviewed and piloted to ensure their comprehensibility in the local context:

- *Psychosocial wellbeing* will be measured using the 9-item Personal Wellbeing Index (PWI) [30]. The personal wellbeing index includes several subscales. Most relevant to this study are the community connectedness and sense of safety subscales. The Spanish version of the full PWI has been validated in Spain where it displayed adequate internal construct validity and excellent internal consistency (α = 0.88) [31]. While no validation studies of the adult PWI have been conducted in Latin America, the adolescent version has been found to display good psychometric properties [32]. Furthermore, an assessment of the PWI’s performance in a multi-country study found evidence of configural measurement invariance and partial metric and scalar invariance across 26 countries [33]. The PWI has also demonstrated good internal consistency among refugee and migrant populations (α = 0.83) [34].
- *Psychological distress* will be measured using the 6-item Kessler-6 [35]. The Kessler-6 has been used as a measure of psychological distress in Panamá and Ecuador, including during the COVID-19 pandemic [36, 37]. Although it has not been validated specifically in Ecuador or Panamá, it has demonstrated good psychometric properties in Latin America as well as in refugee and migrant populations [34].
- *Coping* will be measured using the 28-item Brief COPE [38]. The Spanish Brief COPE has been used to measure coping in previous studies in Latin America and has been found to be associated with other constructs of interest to this study (e.g., psychological wellbeing, social support) [39, 40]. Psychometric analyses of the Spanish version of the Brief COPE identified a unique factor structure that. For example, Morán and colleagues identified a single factor that includes both the emotional and instrumental support items, a single factor that included the active coping and planning items, and did not identify an acceptance subscale [41].
- *Social support* will be measured using the 3-item Oslo Social Support Scale (OSS-3) [42]. The Spanish OSS-3 has been applied in Spain [43], but we did not find evidence that it has been previously used in Latin America. It has also been used in research among immigrant and refugee populations and found to correlate with psychological distress [44, 45].
- *Functional impairment* will be measured using the 12-item World Health Organization Disability Assessment Schedule (WHODAS) [46]. The WHODAS has been previously applied in Ecuador in clinical samples of people with psychiatric and neurological disorders [47, 48]. Validation studies among Ecuadoran populations have reported good internal consistency (α = 0.81) and high convergent validity of the WHODAS when correlated with other measures of disability or mental health problems [47].

### Service-level measures

We will evaluate several service-level outcomes through routine monitoring including participant engagement and retention, safety, and usability.

- *Participant engagement and retention* will be assessed using routine session monitoring forms completed by the facilitators capturing attendance and notes from each intervention session.
- *Safety* will be evaluated as the number of adverse events detected by the research team and reported on an ongoing basis to study investigators and institutional review boards throughout the course of the feasibility trial. We will also inquire about safety through semi-structured interviews with facilitators during the process evaluation.
- *Usability*, which is defined as ‘the extent to which an intervention can be used to achieve specified goals with effectiveness, efficiency, and satisfaction,’ using the 10-item Intervention Usability Scale [49]. The facilitators will complete the Intervention Usability Scale in relation to their experience delivering *Entre Nosotras* after the final session of the intervention. The Intervention Usability Scale was adapted from the commonly used System Usability Scale [50, 51], and has displayed a two-factor structure measuring whether a psychosocial intervention is (1) learnable; and (2) usable [49].

### Implementation-level measures

We will evaluate a range of implementation outcomes pertaining to both the intervention and research procedures.

#### Appropriateness of intervention and research procedures

- We will consider the proportion of those screened who are *eligible* and *sensitivity to change* of our study outcome measures as indicators of appropriateness (i.e., how well aligned our eligibility criteria and outcome measure selection are to the profile and needs the target population).
- We also consider *intervention fidelity* as an indicator of appropriateness. As members of the community trained to deliver this adaptable and flexible intervention, the facilitators may deviate from the manualized intervention to improve the fit of the intervention to the participant’s needs. Intervention fidelity will be evaluated using two methods. A member of our research team will observe at least two sessions and externally assess fidelity using a checklist that indicates whether each activity within a session was completed well, could be improved, or was not implemented. At the end of each session, the facilitator pair reviews the session and for each activity documents whether it was completed and, if so, whether it could be improved; any difficulties they experienced with the activity that may have influenced whether and how it was implemented (e.g., fit/appropriateness); things that worked well (if implemented); or ways in which the activity could be improved.
- During the process evaluation, facilitators and participants will be asked open-ended questions related to the *appropriateness of the intervention and research procedures*. These questions will be informed by the Johns Hopkins Dissemination and Implementation Science Measure [52].

#### Acceptability of intervention and research procedures

- *Acceptability* will be measured by asking participants and facilitators open-ended questions about their satisfaction with the intervention, whether they liked attending or delivering *Entre Nosotras* sessions, and whether the components of the intervention made sense and were useful during the process evaluation interviews. We will also ask about safety as an indicator of poor acceptability.

#### Feasibility of intervention and research procedures

- Feasibility of research procedures will include examining the *rate of recruitment*, whether *randomization* produced balanced groups within site, study *attrition*, and *protocol deviations* assessed using routine study monitoring forms.
- We will examine whether *contamination* occurred using data from the externally-rated fidelity assessments. We will consider any application of Doing What Matters in Times of Stress material in the standard *Entre Nosotras* intervention or the omission of Doing What Matters in Times of Stress material in the enhanced *Entre Nosotras* intervention as evidence of contamination.
- We will explore the *construct validity, internal consistency, and sensitivity to change* of measures of psychosocial wellbeing, psychological distress, coping, social support, and functioning to determine whether they may serve as suitable outcome measures for the definitive trial.
- Feasibility of intervention procedures will focus on whether facilitators are able to achieve the competencies necessary to deliver the *Entre Nosotras* intervention. *Facilitator competency* will be assessed by a member of the research team at the end of training and during the sessions that are observed for the fidelity assessments using a subset of items from the Enhancing Assessment of Common Therapeutic factors (ENACT) rating scale. Specifically, we selected six items from the ENACT that are relevant to the non-clinical nature of the *Entre Nosotras* intervention: (1) non-verbal communication and active listening: eye contact, facial expression, body language, and gestures; (2) verbal communication skills: open-ended questions, summarizing, and clarifying statements; 3) rapport building and self-disclosure; (4) exploration, interpretation, and normalization of feelings; (5) demonstration of empathy, warmth, and genuineness; and (6) elicitation of feedback when providing advice, suggestions, and recommendations. These items are measured on a 3-point scale indicating whether the facilitator needs improvement, partially demonstrated this competency, or demonstrated this competency well. Each facilitator in the pair is rated separately by the external rater.
- During the process evaluation, facilitators will be asked open-ended questions about the *feasibility* of implementing *Entre Nosotras* including general intervention feasibility, *adoptability* within the study contexts, and *potential for scalability and sustainability*. Participants and facilitators will also be asked to identify *barriers and facilitators* to implementation as well as *strategies* for overcoming barriers and leveraging facilitators.

### Sample size

At least two groups (range 2–4) will be implemented per study community. We aim to enroll up to 220 women (10 women per group). A summary of the target sample size (participants, groups) by study site and community is provided in Table 3. This sample size is comparable to prior feasibility trials of group psychosocial interventions and will enable us to sufficiently pilot the study procedures and evaluate the relevance, acceptability, and feasibility of the intervention and trial design [53, 54]. A sample size of 220, is expected to provide sufficient power to evaluate the construct validity and internal consistency of the study outcome measures in a confirmatory factor analysis under most scenarios (e.g., number of parameters and magnitude of loadings) [55].

**Table 3:** Sample size by site and community

Site	Communities (***unit of randomization)***	Number of groups	Number of participants
Guayaquil, Ecuador	• La Florida • Mapasingue and Martha de Roldós	7	70
Tulcán, Ecuador	• Julio Andrade • San Pedro de Huaca • Santa Martha de Cuba and San Luis	7	70
Panamá City, Panamá	• Arraijan • La Chorrera • Panamá City • San Miguelito	8	80

### Data management

All data entered electronically will be range checked to ensure valid values. Any manually entered data will be double entered by two independent research assistants and then range checked for quality. Qualitative data will be audio recorded and then transcribed by a research assistant. All data are coded (de-identified) and stored on a secure server hosted by HIAS to which only the research team has access.

### Planned analyses

#### Quantitative data analysis

##### Descriptive analyses of baseline data

We will use descriptive statistics to characterize quantitative indicators of relevance, acceptability, and most indicators of feasibility. Descriptive statistics will include mean with standard deviation and/or median with interquartile range for continuous variables. Categorical variables will be displayed using frequencies and proportions. We will explore the distribution of demographic and psychosocial characteristics across the study communities and intervention conditions to characterize heterogeneity across sites and identify any major baseline imbalances between study conditions. We will calculate effect sizes to explore the magnitude of baseline differences between study conditions (Cohen’s *d* for continuous variables, Φ for categorical variables) and estimate the significance of this difference using mixed effects models accounting for clustering within communities and sites.

##### Psychometric analyses of baseline data

To evaluate the performance of participant-level outcome measure we will estimate the internal consistency, internal construct validity, and external construct validity (i.e., convergent) of these outcome measures. Internal consistency will be estimated using Cronbach’s alpha. Internal construct validity will be evaluated through confirmatory factor analysis and examination of model fit using Comparative Fit Index (CFI), Tucker-Lewis Index (TLI), root mean square error of approximation (RMSEA), and standardized root mean squared residual (SRMR). We will estimate the correlation among these participant outcome measures to investigator external construct (i.e., convergent) validity.

##### Psychometric analyses of baseline and follow-up data

We will estimate sensitivity to change in participant outcome measures using mixed effects models within individuals, communities, and sites (i.e., within-group changes).

### Qualitative data analysis

Process evaluation interviews will be transcribed and then coded and analyzed in Spanish using the Constant Comparative Method [56]. Within the primary domains of relevance, acceptability, and feasibility, the research team will develop concepts from the data through an iterative coding process. After a set of interviews, the research team will review and code the transcripts. They will discuss emerging themes and identified gaps to be further explored in the next set of interviews. This cycle will continue until the interviews are complete and the researchers agree they have achieved theoretical saturation [57]. The research assistants will then use the final codebook and review all transcripts to identify any overlooked concepts that contribute to themes that emerged at later stages of data collection and analysis.

### Ethics

All study procedures were reviewed and approved by the Institutional Review Board (IRB) at Columbia University Irving Medical Center (USA), Universidad de Santander (Panamá) and Universidad San Francisco de Quito (Ecuador).

#### Informed consent

Informed consent will be obtained by research assistants who completed training in human subjects research ethics prior to beginning the study [58]. Participants must provide verbal consent prior to screening. If eligible, participants then must provide written consent for enrollment prior to beginning the baseline assessment. Participants who are selected to complete the additional process evaluation interview are asked again to provide consent, including permission to audio record the interview. At all phases of the informed consent process and the research study, participants will be reminded that refusal to participate will not impact their ability to access services through HIAS or other agencies.

#### Harms

Data collection may be associated with minimal emotional discomfort due to the discussion of sensitive topics such as protection risks and psychosocial problems. We do not expect that the intervention or the assessments will lead to significant increases in distress. However, members of our research team are trained to identify acute signs of distress and to respond in a supportive manner and/or provide them with referral resources. At least one member of each of our research teams is a clinical psychologist and able to manage emergency situations. If participants require additional mental health or protection services, they will be referred to HIAS’ program staff in these sectors, respectively. HIAS has existing referral protocols in place to manage protection risks and mental health challenges.

We will adhere to local and national guidance regarding social distancing when completing the interviews and will not implement any in-person activities that do not comply with COVID-19 guidance. Participants who complete interviews remotely will be provided with a top-up on their airtime/data to cover the costs of communication during the interview. Participants who complete interviews in person will be reimbursed for transportation costs and provided a small snack.

#### Confidentiality

The intervention sessions will be conducted in groups, which increases the risk of breaching confidentiality. Facilitators will be trained to emphasize the importance of not sharing information about other women with people who are not part of the group. To preserve the confidentiality of women, we will ensure that efforts are made to protect the data, that the identifiable information collected is minimal, and that data collection forms are coded and do not include personally identifiable information.

#### Post-trial care

If we do not detect any risk or harms associated with the stress management component of the intervention, participants in the standard *Entre Nosotras* condition will be given a copy of the Doing What Matters in Times of Stress guide at the end of the intervention period. Overall, the trial is minimal risk. Referrals will be made to HIAS services, as needed, throughout the study period.

#### Protocol amendments

There are currently no protocol amendments to report. Any future amendments will be reported to the IRBs and clinicaltrials.gov.

### Declaration of interests

The authors declare no competing interests.

## Discussion

The results of this feasibility trial will be used to determine whether we should progress to a definitive, fully powered trial evaluating the effectiveness and implementation of the *Entre Nosotras* intervention with and without a stress management component. Furthermore, this feasibility trial will provide preliminary evidence of the feasibility of using comparative effectiveness trials to optimize community-based psychosocial interventions in complex settings. If there are qualitative or quantitative indicators that suggest problems with relevance, acceptability, and/or feasibility of the intervention or study design, we will modify these procedures. We will also examine the validity and reliability of measures of psychosocial wellbeing, psychological distress, coping, social support, and functioning in Ecuador and Panamá as well as among migrant and host community women.

This feasibility trial and potential definitive trial to follow will advance the evidence for community-based psychosocial interventions in humanitarian settings. Consistent methodological challenges related to evaluating these interventions reveal the need for innovative interventions and evaluation approaches to fill this gap in evidence. The *Entre Nosotras* intervention aims to test an adaptable intervention model to which culturally specific and standardized components can be integrated to fit the population needs and operational context. The comparative effectiveness design may provide a foundation for future studies to disentangle the mechanisms by which specific intervention components improve psychological and social outcomes through adaptive and optimization study designs. Furthermore, this flexible intervention design and implementation approach may enable tailoring to different settings and understudied populations (e.g., gender, racial, and ethnic minorities) who may be underrepresented in the current study [59–61]. *Entre Nosotras* represents a novel, adaptable community-based psychosocial intervention that aims to empower migrant and host communities to collectively generate strategies and identify resources to improve the social and psychological dimensions of wellbeing among women in their community.

## Data Availability

Not applicable

## Abbreviations

ACT: Acceptance and Commitment Therapy
CFI: Comparative Fit Index
ENACT: Enhancing Assessment of Common Therapeutic factors
IRB: Institutional Review Board
IUS: Intervention Usability Scale
K-6: Kessler-6
MHPSS: Mental health and psychosocial support
OSS: Oslo Social Support Scale
PWI: Personal Wellbeing Index
RMSEA: Root mean square error of approximation
SRMR: Standardized root mean squared residual
TLI: Tucker-Lewis Index
WHODAS: World Health Organization Disability Assessment Schedule
